# Sinus Floor Elevation with Modified Crestal Approach and Single Loaded Short Implants: A Case Report with 4 Years of Follow-Up

**DOI:** 10.1155/2017/7829179

**Published:** 2017-12-18

**Authors:** Michele Perelli, Roberto Abundo, Giuseppe Corrente, Carlo Saccone, Paolo G. Arduino

**Affiliations:** ^1^Private Practice, Turin, Italy; ^2^CIR-Dental School, Department of Surgical Sciences, University of Turin, Turin, Italy

## Abstract

Tooth extraction is usually followed by bone reduction. In the maxillary posterior region, this remodelling combined with sinus pneumatisation and periodontal defects may lead to a reduced basal bone height available for implant placement. Sinus floor elevation can be performed with different surgical techniques. Crestal approach has demonstrated to be effective, less invasive, and associated with a reduced morbidity. This article reports a modified sinus floor elevation by means of rotary, noncutting instruments, addition of xenograft, and 2 short-threaded implant placements. The aim of the study was to evaluate the implant's success and intrasinus radiographical bone gain after 4 years of functional loading. The premolar implant site presented a starting basal bone height of 6 mm, while the molar site was of 2 mm. In the first surgical step, sinus floor elevation was performed mesially and the implant was inserted, and distally only sinus floor elevation was performed. After 6 months, the mesial implant was uncovered and the second implant was inserted; 4 months later, the second fixture was uncovered, and both fixtures were loaded with single provisional screw-retained crowns and later with single screw-retained porcelain fused to metal crowns. Implants integrated successfully, and crestal bone remodelling did not exceed the smooth collar. Bone gain was 3 mm for the mesial implant and more than 5 mm for the distal one.

## 1. Introduction

After tooth extraction, a physiological alveolar bone reduction may be expected in the horizontal and vertical dimensions [1]. In the maxillary posterior region, this shrinkage may lead to an inadequate bone height to place an implant [2–5].

Various surgical techniques, as well as new implant surfaces, have been developed in attempts to solve these problems [6–10], but there is still lack of evidence regarding which technique is advantageous or superior to the others [10]. Implant placement can be predictable performed simultaneously with grafting when residual basal bone height is 5 mm or more, or it can be performed in a second surgical procedure when the bone available is less than 5 mm [11–13]. Sinus floor elevation can be obtained by means of opening a lateral window on the sinus wall [14], with clinical positive results [15, 16], but with increased morbidity related to complications, higher costs, and increased risk of infections [17–21]. The osteotome crestal approach, first described by Tatum and then modified by Summers [6, 8, 9, 22–24], is less invasive. In this technique, local sinus elevation is achieved through the crestal bone by means of osteotomes, piezosurgery, sonic [25], or rotary instruments and, if required, by adding grafting material to indirectly elevate the sinus membrane with the aim of encouraging new bone formation, thus increasing implant osteointegration. The necessity of grafting material, and its composition, has being questioned. After sinus membrane is elevated, an anatomical compartment is created which fills with a blood clot. If Schneiderian membrane is not perforated, this clot is protected and acts as a matrix for bone regeneration. Various grafting materials have been proposed to maintain this compartment stable against the membrane's collapse, due to sinus pneumatisation, and to promote new bone formation. Among these, collagen sponge is the faster to be resorbed, while xenograft demonstrated to be stable with no or partial resorption over time [26, 27]. The use of short implants (≤7 mm long) [28] in the posterior maxilla is well documented, and it has been demonstrated to be successful over years [29–32].

The aim of this study was to evaluate, at 4 years, the clinical success and radiographical bone gain in the treatment of a posterior atrophic maxilla, using short-threaded implants by means of crestal sinus floor elevation with collagen sponges in combination to implant placement when possible and collagen sponges plus xenograft when a staged approach was required.

## 2. Case Presentation

**Figure 1:**
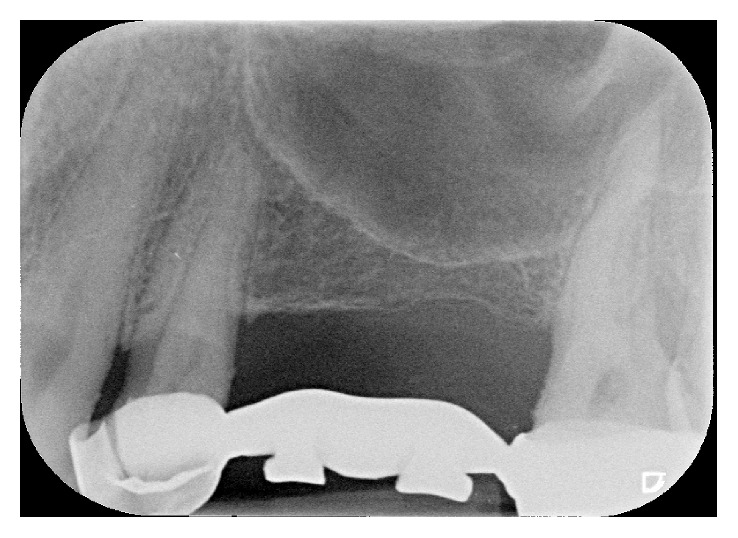
Maxillary left posterior PFM bridge must be replaced. Two implant-supported crowns need to be placed. In both surgical sites, reduced basal bone height is available.

**Figure 2:**
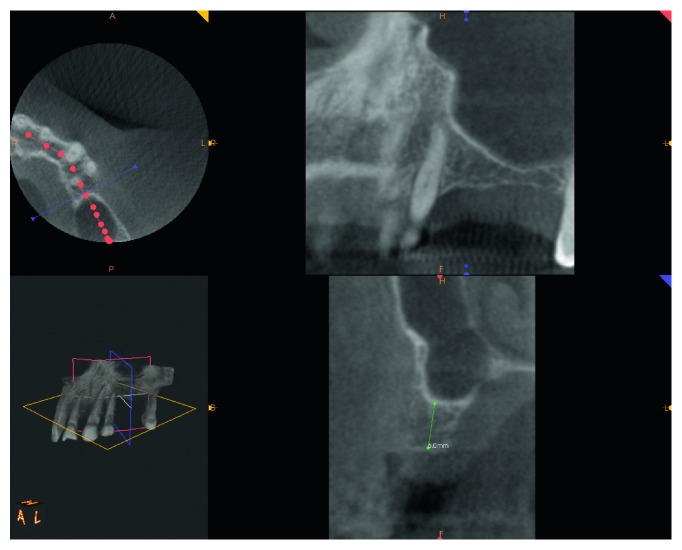
CT scan pre-op showing 6 mm Bbh at mesial site.

**Figure 3:**
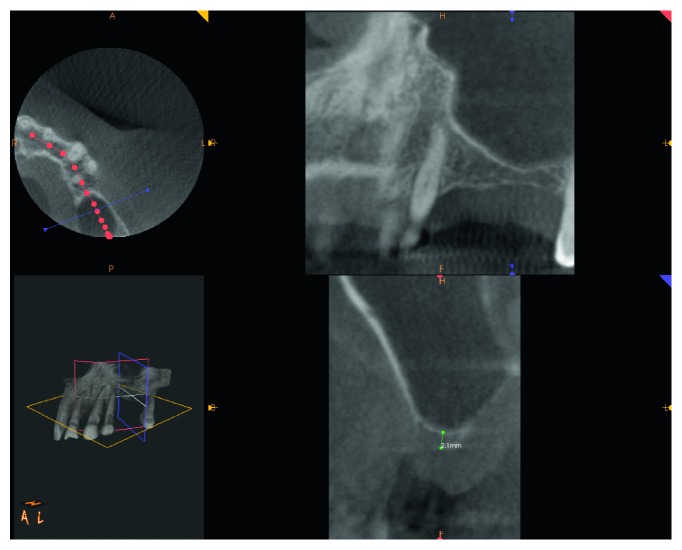
CT scan pre-op showing 2 mm of Bbh at distal site.

A female patient, 65 years old, nonsmoker, presenting a fixed partial denture to be restored in the posterior left maxillary region, with reduced basal bone height, was recruited. She presented no general or local surgical contraindications and signed an informed consent. This study was conducted in accordance with the Declaration of Helsinki (2002). Baseline measurements of the available basal bone height (Bbh) were recorded with digital radiography, obtained using the paralleling technique by means of Rinn's holder, and with computed tomography (CT) scan. Two teeth were needed to be replaced with implant-supported crowns in the second premolar and first molar areas. The first surgical site presented a Bbh of 6 mm, while the second a Bbh of 2 mm (Figures 1–3). After local anaesthesia, using articaine with adrenaline (1 : 100,000), a full-thickness mucoperiosteal flap was elevated.

Osteotomies and sinus membrane elevation were performed using SCA system (Sinus Crestal Approach, Neobiotech, South Korea), according to the manufacturer's protocol [33].

The first cutting drill (diameter of 2.0 mm) was used with a drill-stop positioned 1 mm shorter than the expected Bbh. Later, the first S-Reamer (diameter of 2.8 mm) was used at the expected Bbh, progressing until the cortical sinus floor was perforated. Then, a second 3.2 diameter S-Reamer was used at the same working length. These two reamers have a noncutting flat surface with an S-shaped area which is able to remove the bone beneath the sinus floor without tearing the sinus membrane. A proper depth gauge was inserted carefully to check the integrity of the membrane and the correct osteotomy. The graft was pushed with a condenser with the stop at the exact Bbh (not protruding into the sinus); finally, the bone spreader was applied in order to spread particulate graft material laterally and homogeneously around osteotomy below the membrane. This last device was inserted with a stop 1 mm into the sinus cavity.

**Figure 4:**
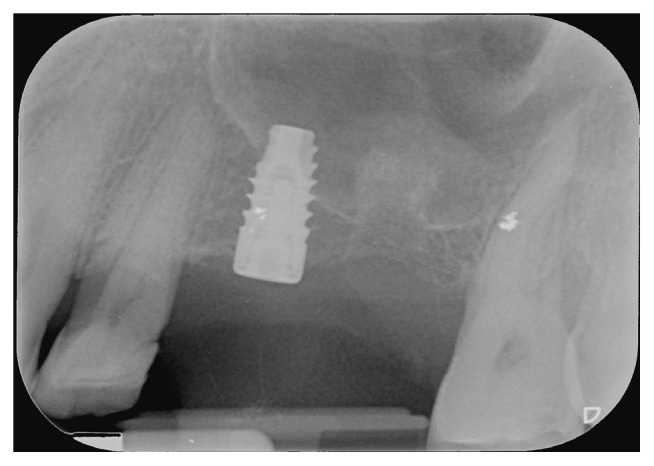
Crestal sinus floor elevation was performed. In the premolar site, collagen sponges were placed, and a 4.25 × 8.5 mm long implant was inserted. In the molar site, a “future site” elevation was achieved with collagen sponges deeply together with a xenograft at the base of the sinus and into the osteotomy.

**Figure 5:**
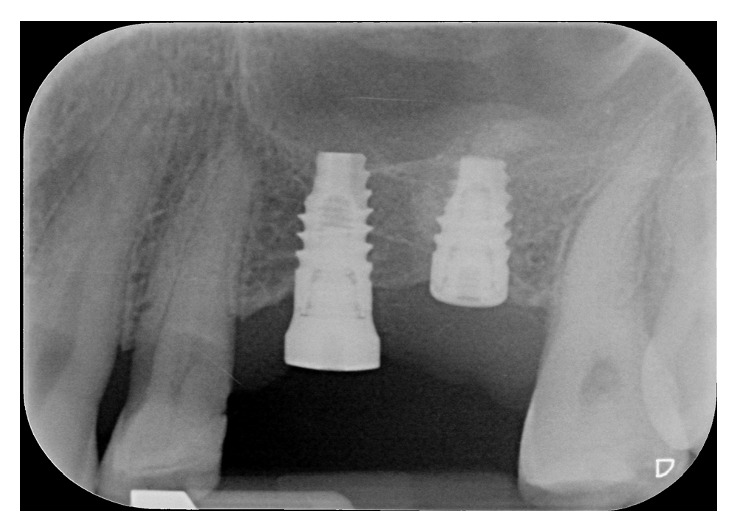
After 6 months, the mesial implant was uncovered, and a short implant was positioned in the molar site with an ulterior crestal sinus floor elevation. Note the new sinus floor level on the apex of the implant #25 and the displacement of collagen and basal bone on the top of the distal fixture.

**Figure 6:**
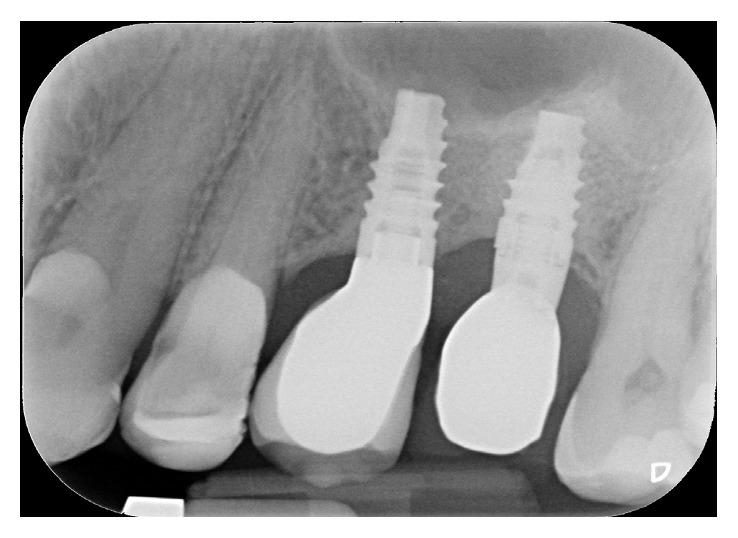
4 years' follow-up radiography. Sinus floor level is maintained on the top of both implants' apex and appears well corticalized. Crestal bone remodelling occurred around implant #26, not exceeding the smooth collar.

In the premolar site, sinus floor elevation was performed with equine collagen sponges (Condress, Smith & Nephew, Agrate Brianza, Italy), together with a short-threaded Premium Straight 4.25 mm × 8.5 mm implant (Sweden and Martina, Due Carrare, Padova, Italy), with the smooth collar placed at the bone level. Differently, in the first molar site, sinus floor elevation was performed by applying collagen sponges together with xenograft (Bio-Oss, Wolhusen, Switzerland) at the basal bone level and in the osteotomy site, to provide more stability to the collagen (Figure 4). Flaps were replaced and sutured, and a submerged healing was obtained. The patient received antibiotic therapy: 1 g of amoxicillin starting from the day of surgery (1 h before surgery), twice a day, for 5 days; ibuprofen 600 mg was prescribed to be taken if needed, and chlorhexidine spray to be used 3 times daily for 15 days. Sutures were removed after 15 days. No intra- or postoperation complications occurred. An intraoral radiography was taken at 3 months. After 6 months, a second surgical approach was done; the premolar implant was uncovered and a short Premium Straight 4.25 × 7 mm implant (Sweden and Martina, Due Carrare, Padova, Italy) was placed in the first molar area. Also in this case, osteotomy was done with SCA technique and collagen sponges were positioned to displace the Schneiderian membrane (Figure 5). The mucoperiosteal flap was repositioned and sutured, assuring a covered healing of the molar implant, while the mesial fixture was uncovered with a healing cap of 4 mm height. Drugs were prescribed as previously noted, and the implant was left to heal for 4 months. After that period, the molar implant was unscrewed, and both fixtures' osteointegration was checked with a reverse torque of 30 Ncm. Implants resulted correctly integrated; transfers were positioned and a polyvinyl siloxane impression was taken. Provisional screw-retained single crowns were delivered, and, after 4 months, definitive porcelain fused to metal screw-retained single crowns was positioned. Control radiographies were taken after 3 months from the first surgery, after 4 months from the second surgery, at the time of provisional crown setting, at 12 months, and then yearly (Figure 6).

**Figure 7:**
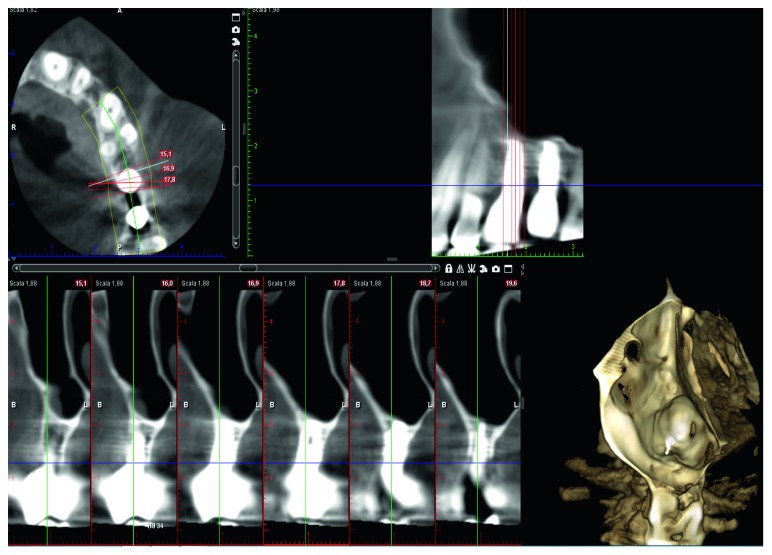
Implant site #25 CT scan at 4 years.

**Figure 8:**
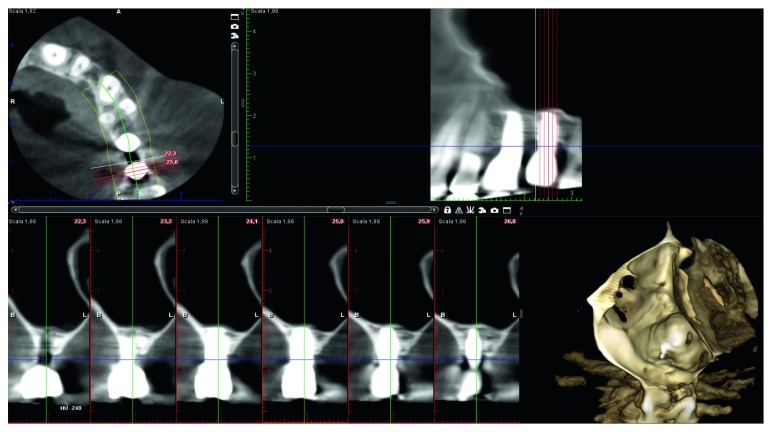
Implant site #26 CT scan at 4 years.

No mechanical or prosthetical complications were reported. Radiographs were taken at 3, 6, 10, and 14 months starting from the baseline (Figures 6–8). Initial Bbh was almost 6 mm for the first site and 2 mm for the second site. Immediately after the surgery, the implant # 2.5 presented sinus floor level at the 4th thread mesially and at the 3rd thread distally. After 10 months, all the implant surface was radiographically covered with bone, which means a radiographical bone gain of 3 mm mesially and 5 mm distally. The molar surgical site presented a Bbh of 2 mm at baseline, with a flat bone crest. After two-step sinus elevation, a 7 mm long implant was radiographically completely covered by bone, exceeding also the apex (7 mm of bone gain). The radiographical bone gain between the fixtures was almost 5 mm. The xenograft was clearly noticed in all radiographs, while the previous collagen-filled space was replaced with more bone-like image. Crestal bone remodelling occurred only on the distal implant, but not exceeding the smooth collar. Both implants were restored with single screw-retained crowns, and no mechanical complications occurred in 4 years.

## 3. Discussion

In accordance with the 1996 consensus conference on sinus lifting [34], the suggested treatment of the atrophic posterior maxilla is sinus floor elevation with a lateral approach plus a xenograft. Moreover, a staged approach is recommended when Bbh is insufficient to guarantee the primary stability of the implant. Unfortunately, this technique presents complications such as infections, graft failure, membrane perforation, and bleeding from the lateral trap door. Crestal approach is demonstrated to be a safe and predictable technique [26, 35, 36] with implant survival rates comparable with lateral approach [37].

In addition, if more implants must be placed, sinus floor elevation performed in different adjacent sites may hesitate in a wider membrane elevation, as described by Reiser et al. [38].

The presence of a xenograft is not fundamental to obtain de novo bone formation and implant stability [39, 40]. Performing crestal sinus floor elevation with collagen sponge simultaneously with implant placement may hesitate in endosinus bone gain limited to the height of the implant apex, because the Schneiderian membrane might collapse on it [41], as demonstrated by the mesial implant. To increase bone formation, the membrane must be maintained in an elevated position [42]. As demonstrated by the second implant, when a staged approach is done, the presence of a xenograft to sustain the collagen sponge (as well as adjacent implant) is capable of maintaining the membrane displaced. When the implant is positioned, a new bone mixed with xenograft is again dislocated apically, thus determining an ulterior membrane elevation. This displacement hesitated in a sinus floor corticalization, exceeding the apex of the second implant with a great interimplant bone gain. Implants were loaded with single screw-retained crowns in order to easily deal with biological or prosthetic complications and improve the patient's compliance and oral hygiene.

Recent metaanalysis reported success of short (7 mm long) implants inserted in the posterior atrophic maxilla loaded with single crowns [43], with minimal crestal bone remodelling, reducing the risk of cement retention, difficult oral hygiene, and prosthetic complications or failures.

This case report describes sinus floor elevation with crestal approach and short-threaded implant positioning in the treatment of a posterior athropic maxilla. When Bbh was more than 5 mm, it was possible to elevate and to place simultaneously the implant with the addition of collagen sponges. By contrast, when Bbh was less than 5 mm, with no sufficient implant primary stability, a staged approach has been performed using collagen sponges deeply, against the Schneiderian membrane, together with a xenograft at the basis of the sinus floor to contraact membrane collapse and sustain the collagen. When the implant was placed in this area, an ulterior elevation was performed. Implants demonstrated to be stable at 4 years' control, as well as bone levels (both crestal and sinus floor) (Figures 7 and 8).

The results of this study must be confirmed with larger sample clinical trials with a longer follow-up.
